# Combining Inter-Subject Modeling with a Subject-Based Data Transformation to Improve Affect Recognition from EEG Signals

**DOI:** 10.3390/s19132999

**Published:** 2019-07-08

**Authors:** Miguel Arevalillo-Herráez, Maximo Cobos, Sandra Roger, Miguel García-Pineda

**Affiliations:** Departament d’Informàtica, Universitat de València, Avda. de la Universidad, s/n, 46100-Burjasot, Spain

**Keywords:** EEG, arousal detection, valence detection, data transformation, normalization

## Abstract

Existing correlations between features extracted from Electroencephalography (EEG) signals and emotional aspects have motivated the development of a diversity of EEG-based affect detection methods. Both intra-subject and inter-subject approaches have been used in this context. Intra-subject approaches generally suffer from the small sample problem, and require the collection of exhaustive data for each new user before the detection system is usable. On the contrary, inter-subject models do not account for the personality and physiological influence of how the individual is feeling and expressing emotions. In this paper, we analyze both modeling approaches, using three public repositories. The results show that the subject’s influence on the EEG signals is substantially higher than that of the emotion and hence it is necessary to account for the subject’s influence on the EEG signals. To do this, we propose a data transformation that seamlessly integrates individual traits into an inter-subject approach, improving classification results.

## 1. Introduction

Affect recognition has been an active topic of research for the last two decades, and attempts have been made to detect emotions from many different sources of information, including text [1], facial expressions [2], speech [3], physiological signals [4,5,6] or interaction data [7], among others.

Electroencephalography (EEG) signals were initially used in medicine to diagnose a diversity of disorders and pathological conditions, such as epilepsy [8,9], alcoholism [10,11], detection of suicidal ideation [12] or monitoring the depth of anesthesia [13]. However, the large quantity of information that EEG signals encode about the subject has motivated their use in other application areas, such as biometric recognition [14,15], gender identification [16] and emotion detection [17,18].

Previous neuropsychological studies [19] have shown a relation between emotions and the electrical activity of the brain, and reported on EEG correlates of emotions [19]. This relation has motivated a large number of attempts to detect emotions by processing EEG signals, sometimes in combination with other sources of information (e.g., [20]). However, EEG signals are relatively complex, and affected by physiologic and extraphysiologic artifacts such as eye movement, pulse, respiration or measurement equipment. Therefore, there is an intrinsic difficulty associated with making this relation explicit. This includes the use of appropriate signal processing methods to cancel undesired artifacts [21,22]; the extraction and selection of the most informative features and channels [23,24]; and the development of techniques that are able to detect patterns that can be linked to specific emotional states (e.g., [25]).

Previous works in the field of psychology suggest that there are significant differences in the way individuals feel and express emotions [26]. Many typical setups use a set of training samples to build a general, subject-independent (inter-subject) model, which is shared by all users (e.g., [27,28,29,30]). In this case, a single model is built by considering all data as if it were coming from the same subject, without taking the user’s particularities into consideration. Despite the high prediction rates obtained in some cases, these can be significantly improved by using individual models adapted to each user [30,31,32]. However, subject-dependent (intra-subject) approaches suffer from two severe drawbacks. First, they require the collection of a large amount of data to adequately model the relation between the EEG signal and the emotion for each person. Second, they cannot be used for unseen subjects as they only use data related to the particular individual. These two drawbacks make the approach impractical in many cases.

In this paper, we first study the suitability of intra-subject and inter-subject modeling approaches in an EEG-based affect recognition context, by analyzing the available data in three public databases, namely the Database for Emotion Analysis using Physiological Signals (DEAP) [33], MAHNOB-HCI [34] and DREAMER [35]. The analysis performed clearly indicates that the contribution of the subject to the EEG signal is far larger than the effect of the emotion, hence limiting the applicability of inter-subject models and suggesting a better behavior of subject-dependent models that only use training data associated with the same subject. An in-depth analysis using the DEAP dataset also reveals that many positive results for subject-independent models reported in some previous works may in part be due to the use of imbalanced datasets. As a second and more important contribution, we propose an approach that combines an inter-subject model with a subject-based normalization of the EEG signals, making it possible to effectively generate a single model, which is valid across the entire population. This approach integrates data related to personality traits into the model, encoding a person’s individuality in feeling emotions without affecting data capturing needs. The gains achieved open the door for using a single model for unseen subjects, which can be progressively adapted as more personalized data are gathered.

This paper is structured as follows. First, related previous work is described in Section 2, covering both modeling approaches and existing public databases. Then, in Section 3, the three repositories considered are analyzed by computing an embedding that reveals key issues related to the topological structure of the data. After, we present our proposal to partially cancel the subject-related component from the signal to achieve an inter-subject model with comparable performance to typical intra-subject models. This method is evaluated in Section 5. Finally, the main conclusions from this work are presented in Section 6.

## 2. Related Previous Work

### 2.1. Modeling Approaches

Computational methods for affect detection attempt to relate features extracted from certain signals measured on a subject to emotional processes. These features may be captured from, e.g., facial expressions, voice, body language and posture, physiological states, functional Magnetic Resonance Imaging (fMRI), Magnetoencephalogram (MEG) brain signals and/or EEG. In general, machine learning algorithms are used to identify signal patterns that are associated with the expression of different emotions, and to build models that enable the automatic detection of a concrete set of states (see, e.g., [36,37,38] for extensive reviews of the field). These machine learning approaches can be classified as inter-subject or intra-subject. Methods in the first category aim at constructing a model that is valid for all users. Techniques in the second one consider that the appraisal of one’s emotional state is strongly related to personal factors, such as one’s circumstances [39]. Hence, they aim to construct an individual model for each user, generally increasing performance at the cost of increasing data collection needs [40,41].

The prediction of a subject’s emotion/mental state from brain signals has been widely studied, including both EEG and MEG signals [42,43]. In the particular case of EEG, the signals from selected channels are usually pre-processed with noise reduction algorithms and filtering methods to enhance the signal-to-noise power ratio. Feature extraction is then used to determine variables which correlate well with the target emotional states, according to the specific emotional model that is used [19]. Typical feature extraction methods include wavelet transform [44], spectral power features [45], higher order crossings [46], short-time Fourier transform [47], asymmetry index [48] and/or statistical features [49], e.g., mean, standard deviation, variance, quadratic mean, skewness, power or entropy. Finally, a classification method is used to discriminate a particular emotional state from the features. Support Vector Machines (SVM) [47,48,50,51], nearest neighbour classification [45,50], Naive Bayes [50] or Linear Discriminant Analysis (LDA) [52] are examples of an extensive list of methods that are applied in this context.

Most EEG-based emotion recognition studies use Rusell’s two dimensional bipolar emotional model to label and represent emotional states, which is based on valence and activation/arousal [53]. This representation relies on the fact that these two variables account for the major proportion of variance in affect scales. In such models, each emotion is found as a combination of values for valence and arousal, falling meaningfully around the perimeter of the space. The valence dimension represents whether the emotion corresponds to a positive or a negative feeling; and the arousal refers to the level of excitement. The valence/arousal representation was extended to a 3D space in [54], by also considering whether the subject feels controlled or in control of the situation (dominance).

### 2.2. Public Databases

The intensive work in emotion recognition using EEG data has been supported by the existence of a number of public datasets. A first large database is DEAP, which is presented in [33]. DEAP contains EEG and peripheral physiological signals of 32 people who were recorded as each watched 40 one-minute long excerpts of music videos. These were stored along with the levels of arousal, valence, like/dislike, dominance, and familiarity reported by the subjects. The dataset also contains frontal face video for 22 of the participants. In addition, methods and results are presented for single-trial classification of arousal, valence, and like/dislike ratings using the modalities of EEG, peripheral physiological signals, and multimedia content analysis. EEG signals were recorded by using a Biosemi ActiveTwo system. Despite its relatively recent publication, DEAP [33] has been extensively used in the affect recognition field, to evaluate a number of proposals (e.g., [55,56,57]).

Another large database is presented in [34]. In this case, the repository contains data for 27 people, recorded while watching 20 movie fragments and pictures in a very similar setting as in DEAP. In this case, video data are provided for all participants, from six different cameras. The database also contains eye gaze information, as well as other physiological signals (including EEG). Data are stored along with the emotional state reported by the subject, both using emotional keywords and on a scale of valence, arousal and dominance. EEG signals were recorded by using active AgCl electrodes placed according to the international 10–20 system (32 channels).

More recently, a third dataset of similar characteristics as the previous with regard to the EEG data provided has been published [35]. Under the name of DREAMER, this dataset contains EEG data from 23 participants as they watched 18 music videos. The main difference with respect to the previous two databases refers to the type of equipment that was employed. In DREAMER, 128 Hz EEG signals were recorded using an Emotive EPOC system, a device that offers a considerably lower precision than the Biosemi Active Two. Table 1 summarizes the characteristics of these three databases.

Major problematic issues that have hindered the development of practical applications that use EEG signals are related to the cost, time resolution, and complexity of setting up experimental protocols that resemble real-world activities [36]. This has motivated a track of work that focuses on mobile/low cost devices (e.g., [55,58,59]). Although these devices may be less accurate at the signal acquisition phase, they may offer a comparative performance at detecting emotional changes in the subject. This has led other authors to develop their own datasets to validate their results in specific contexts that use low cost devices [55,59]. However, these datasets have not been made public and are hence not usable in other research works.

## 3. Data Analysis

### 3.1. Problem Formulation

Let us assume a set of subjects $S=\{s_{i}\},\;i=1,2\ldots m$. Let us also assume that there exist a set of $n_{i}$ labeled training samples for each subject $\forall s_{i}:T_{s_{i}}=\left\{\left(t_{s_{i},j},l_{s_{i},j}\right)\right\},\;j=1,2\ldots n_{i}$, where $t_{s_{i},j}$ is conveniently represented in a particular feature space F and corresponds to the feature vector for the *j*th sample of subject $s_{i}$, and $l_{s_{i},j}$ refers to the corresponding emotional label.

Current emotion recognition approaches can be classified into inter-subject and intra-subject. In practice, both types of models are typically built by using classification approaches on training data. In general, this training data (the sets $T_{s_{i}},\;i=1,2\ldots m$) consist of a number of labeled entries that relate features to emotions. The fundamental difference between the two approaches is whether the labeled training data refer to a single individual (intra-subject) or to a group of people who are collectively treated as if there were no particularities that make individuals different from each other (inter-subject). In inter-subject methods, a global model which is valid for all users is built, by using the training data $T_{s_{1}}⋃T_{s_{2}}⋃\ldots ⋃T_{s_{m}}$. This is, in fact, equivalent to treating all training data for different individuals as if they belong to the same subject [28,29,30,59]. In intra-subject approaches, an independent model is built for each subject $s_{i}$ [27,31,32,40,41], by considering only training data that belong to that particular subject ($T_{s_{i}}$). The high accuracy achieved by some subject-independent models (e.g., [28,29,59]) suggests that some relations between features and emotions hold for most individuals. At the same time, the usually better prediction performance achieved by intra-subject models [40,41] suggests that the relation between the EEG features and the emotions are, in reality, subject-dependent. Hence, relations between features and emotions can be better established when the user’s particularities are taken into consideration. However, intra-subject models require exhaustive data collection from the same subject to build the model. Furthermore, they cannot be used on previously unseen individuals, unlike with inter-subject models.

### 3.2. Topological Structure of the Data

For the purpose of this work, we replicated feature extraction as described in the original publications describing each database. First, we calculated the Power Spectral Density (PSD) using Welch’s method with a Hamming window of 128 samples and 50% overlapping. The spectral power was averaged over the θ (4–8 Hz), slow α (8–10 Hz), α (8–12 Hz), β (12–30 Hz), and γ (>30 Hz) bands from all electrodes. In addition, we computed the difference between the spectral power of all the symmetrical pairs of electrodes on the right and left hemisphere in the same bands, to measure the possible asymmetry in the brain activities due to emotional stimuli. This yielded 230 features for DEAP and MAHNOB-HCI (32 electrodes × 5 bands + 14 pairs × 5 bands), and 105 features in DREAMER (14 electrodes × 5 bands + 7 pairs × 5 bands), as reported in Table 1.

The resulting features were used to plot a 2-D (two dimensional) map after a space transformation using t-Distributed Stochastic Neighbor Embedding (t-SNE) [60]. t-SNE is an unsupervised dimensionality reduction method that is particularly well suited for the visualization of high-dimensional datasets. t-SNE is capable of capturing and preserving much of the topological structure of the high-dimensional data, while also revealing global structure such as the presence of clusters at several scales [60]. We reduced the data to two dimensions, so that we could easily display and analyze it using a scatterplot.

**Figure 1:**
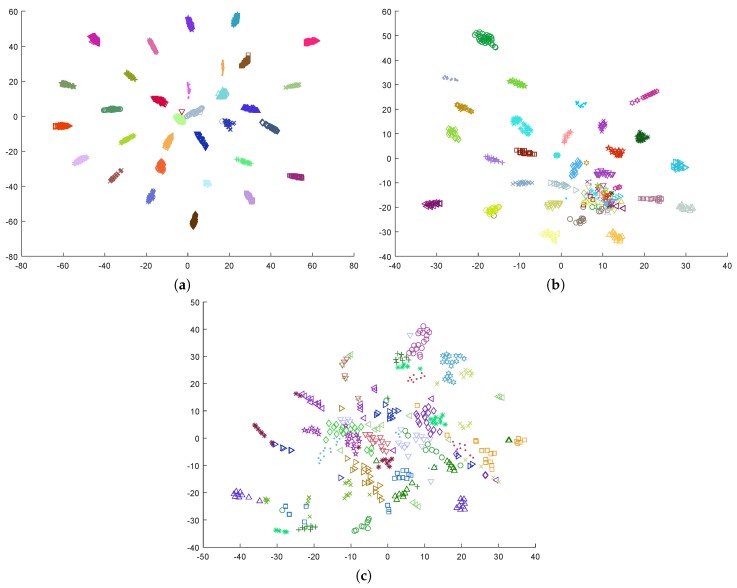
Dimensionality reduction by t-SNE on original data: (**a**) DEAP; (**b**) MAHNOB-HCI; and (**c**) DREAMER. Each subject has been represented with a different colored marker.

Figure 1 shows the result produced by t-SNE method on the three databases used in this work. We plotted samples from each subject using a different colored marker, to easily observe that EEG data samples from the same subject are topologically located close to each other in the 2-D space. These plots reveal that the contribution of the subject to the EEG signal is clearly higher than the effect of the emotion, a fact which has been extensively exploited in biometrics (e.g., [61,62,63]).

Although it seems clear that the topological structure of the maps presented in Figure 1 is not the best for the construction of inter-subject models, other previous works have obtained positive results when applying subject-independent models using a typical classification setting. For example, the affect recognition results reported in [35] refer to accuracies of 0.62 in valence and arousal, using a SVM with a Radial Basis Function (RBF) kernel. However, they used an imbalanced dataset, with a proportion of 56–44% in arousal and 61–39% in valence. Considering Figure 1c, it is possible that the positive accuracy reported is in part due to this fact, rather than to the existence of emotion-evoked specific EEG patterns that are shared by multiple subjects.

## 4. Proposed Approach

### 4.1. Typical Data Transformations

**Figure 2:**
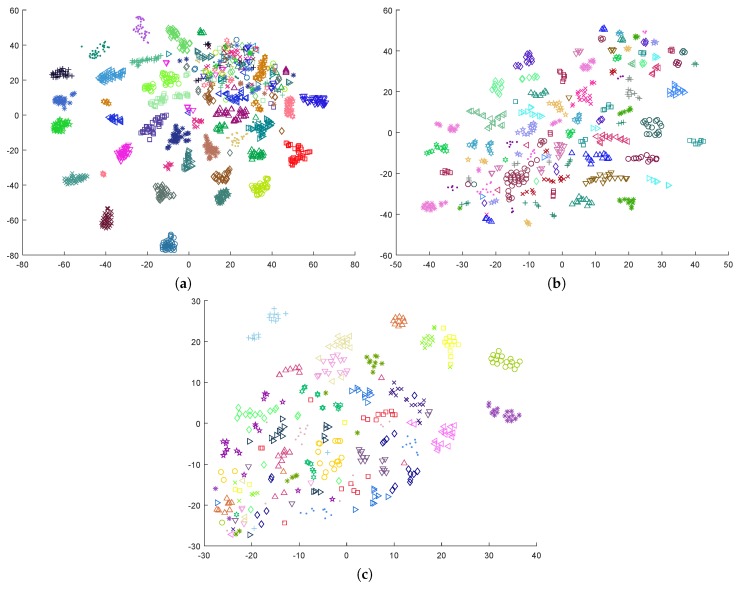
Dimensionality reduction by t-SNE, after normalizing the data by scaling each feature between the maximum and minimum values for the particular subject: (**a**) DEAP; (**b**) MAHNOB-HCI; and (**c**) DREAMER.

The construction of inter-subject models is a harder problem due to the high EEG variability between individuals [64]. The three plots in Figure 1 clearly indicate that classification approaches that use these data would benefit from the removal of the subject’s contribution to the EEG signal. Instead of producing an intra-subject model with personalized data coming from a single individual, the subject’s particularities can be incorporated into an inter-subject global model by normalizing the data from each subject according to a subject-dependent baseline that summarizes the contribution of the individual to the EEG signal. Other previous works have implicitly attempted this by applying a subject-based normalization of the data. For example, in [33,65], the features were normalized for each participant by scaling them between 0 and 1 to reduce inter-participant variability. The effect of this normalization is shown in Figure 2, for the three databases considered in this work. The effect of such a linear normalization on the subject related component is somehow limited and the latent clustered structure of the original data remains, but the lower distance between the clusters suggests that the subject component in the EEG signals has at least been reduced. This fact outlines the potential of subject-dependent normalizations, and suggests that other more elaborated data transformations may be applied to further reduce or eliminate the subject-related component from the EEG signals.

### 4.2. Nonlinear Data Transformation

In particular, and to explore the potential of subject-dependent methods other than a linear scaling, we tested a simple nonlinear transformation of the original data. First, we independently considered each subject, and computed the median value for each feature. Then, the original feature vector was codified as a binary vector of the same size, where components take values 0 or 1 depending on whether the feature value is lower or higher than the median, respectively. More specifically, for any subject $s_{i}$, we considered all feature vectors $t_{s_{i},j},\;j=1,2\ldots n_{i}$ in the set of training samples $T_{s_{i}}$ and computed the median vector ${\tilde{t}}_{s_{i}}$ across each feature. Then, all feature vectors u for the same user $s_{i}$ were transformed according to Equation (1)

(1) $$\hat{u}\left[k\right]=\left\{\begin{array}{cc} 1 & \;u\left[k\right]>{\tilde{t}}_{s_{i}}\left[k\right],\\ 0 & \;u\left[k\right]\leq {\tilde{t}}_{s_{i}}\left[k\right], \end{array}\right.$$

where [k] denotes the *k*th element (feature) of the corresponding vector.

**Figure 3:**
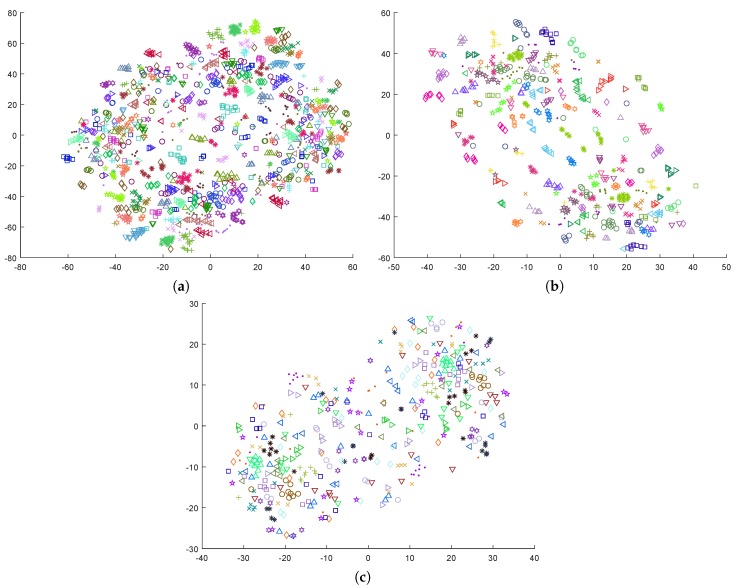
Dimensionality reduction by t-SNE, after transforming the data by binarizing values according to whether they are lower or greater than the median: (**a**) DEAP; (**b**) MAHNOB-HCI; and (**c**) DREAMER.

**Figure 4:**
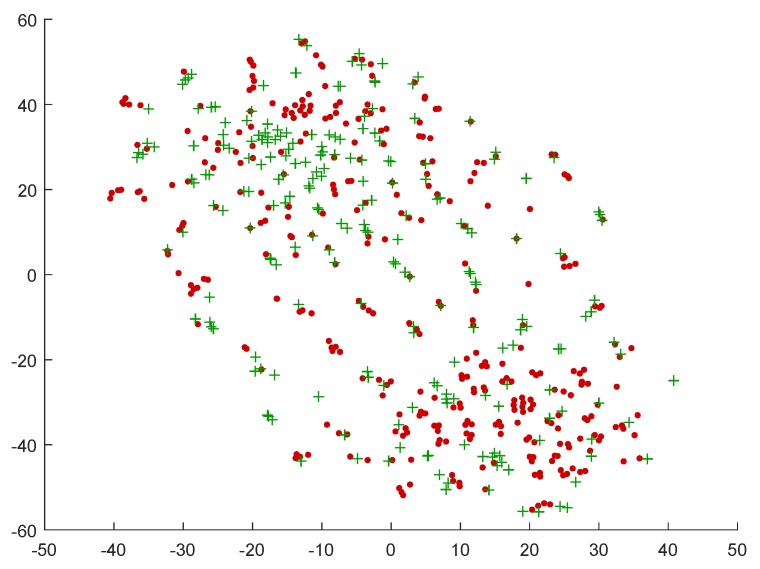
Positive (green plus markers) and negative (red dots) arousal samples in the MAHNOB database, on the representation space produced by t-SNE.

Figure 3 contains the t-SNE representation for the data when this transformation is applied to the entire set. As can be observed, and despite the information loss that is inherent to this operation, the data samples from a same group now appear more sparse, and these plots suggest a more effective reduction of the subject-related component of the signals. A further analysis of the data topology with regard to the labels also revealed a certain level of grouping, more suitable for classification purposes. As an example, Figure 4 shows the positive and negative samples in the MAHNOB dataset in the t-SNE space, according to self-reported arousal levels. An inspection of this plot in relation to the one in Figure 3b suggests that the samples for certain groups of subjects may have been split according to their label.

The proposed transformation allowed us to train the classifier using data from all available subjects, avoiding the small sample case and the need for the personalized training that is typically required when using intra-subject approaches. The only data required by the proposed transformation are the median for each feature, and these can easily be computed and progressively refined from unlabeled data as soon as the EEG capturing device is connected.

## 5. Experimental Results

### 5.1. Improvement on Classification Accuracy

To exhaustively assess the effect of the proposed data transformation, we ran a number of experiments aimed at testing the prediction performance on previously unseen subjects. Results obtained with the proposed data transformation were compared using *z*-score standardization, a typical data normalization commonly used in machine learning contexts. To this end, we computed the mean and standard deviation vectors μ and σ from the samples in the training set, and normalized each feature vector x according to Equation (2).

(2) $$\hat{x}\left[k\right]=\frac{x[k]-\mu [k]}{\sigma [k]}$$

For a comprehensive evaluation, we applied several classification methods, namely SVM with polynomial and Gaussian kernels and Naive Bayes, to be consistent with the previous literature in the field [18,33,34,35]. All experiments were run in a Matlab R2017a environment, using Matlab’s own implementation of the classification algorithms.

All datasets were pre-processed as in [66] to appropriately compare the methods and avoid misleading results caused by different degrees of imbalance in the intra-subject and inter-subject cases. In each database and for each of the labels analyzed (arousal and valence), we randomly selected the same number of samples per class for each user. The number of samples was decided to simultaneously achieve sufficiently populated training sets and minimize the number of subjects that had to be discarded because they did not have sufficient samples in the minority class. Table 2 summarizes the resulting number of users and the samples per user after processing the datasets in this way.

In each dataset, and for every combination of normalization and classification method, we ran 20 experiments per subject. In each experiment, all data for one subject $T_{k}$ were used as the test set, and 90% of the data from the rest of the individuals, i.e., $\left(T_{1}⋃T_{2}⋃\ldots ⋃T_{m}\right)-T_{k}$, were employed for training. As the classes in the three datasets were balanced and had equal importance, the performance was assessed using classification accuracy. This was computed as the proportion of instances that were correctly categorized according to the self-reported binary labels for arousal and valence provided as a ground-truth in each database.

Table 3 compares the classification accuracy when using a typical *z*-score normalization and when the proposed subject-based normalization was applied. To effectively rank the two algorithms according to their general performance, and measure the statistical significance of the results, their classification accuracy was evaluated separately for each test and training pair. With these measurements, a multiple comparison Friedman test [67] was conducted, considering the null hypothesis that the two methods obtained similar results with non-significant differences. This non-parametric test requires computing the average ranks of all methods, which are shown in Table 4, along with the *p*-values and the number of pairwise comparisons that allowed their computation. The *p*-values were calculated using software available from http://sci2s.ugr.es/sicidm [67].

When using a radial SVM or the Naive Bayes classifier, the improvement achieved by the proposed subject-based normalization was always statistically significant with *p*-values below $10^{-3}$ in all cases, which allowed us to reject the null hypothesis. When using a cubic SVM, *p*-values were generally higher, and above 0.05 in one case. Nevertheless, all entries in the table support the performance increase achieved by the proposed data transformation.

As a reference, we also provide in Table 5 the classification accuracy achieved when using an intra-subject model, which was obtained using a different setting. To compute these values, we averaged the results of 100 experiments for each user. In each of these experiments, we selected one positive and one negative sample from the concrete user as the test set, and used the remaining samples for training. This yielded a total of 2×m×100 judgments, with *m* the number of subjects in the pre-processed dataset.

When using a standard *z*-score normalization, it can be observed that the accuracy for intra-subject models was generally better, except in the DREAMER database, which showed very poor results in all cases. This was despite using considerably fewer training data. In general, the accuracy of inter-subject models that use *z*-score standardization remained close to 50% in most cases, a result that is consistent with the data topology shown in Figure 1, in which samples are grouped by subject rather than their emotional label. On the contrary, the intra-subject models showed reasonable accuracies that are consistent with results reported in previous works [18,33,34], ranging from 0.54 to 0.66 in the DEAP and MAHNOB databases.

When using the proposed data transformation, a significant performance improvement was achieved with regard to the *z*-score normalization. The results are clearly outperformed in all cases. On many occasions, the inter-subject model on the normalized data performed better than the corresponding intra-subject model. Even in DREAMER, the data transformation led to a reasonable classification accuracy, close to that obtained in other repositories. Rather than a clear performance advantage, the results reported in Table 3 show a comparable performance between using an intra-subject model and the suggested data transformation. However, the proposed approach can be used for previously unseen subjects despite not having additional data available for that specific individual, and offers a performance which is significantly better than that obtained by using a typical *z*-score normalization.

The behavior reported is consistent with the plots in Figure 1 and Figure 3. When the subject-based data transformation was not applied, the intrinsic subject-dependent component in the signal dominated the data topology, leading to a highly inefficient model for previously unseen subjects. However, the intra-subject model performed reasonably well, as this component equally affected all samples and inherently canceled out. The proposed subject-dependent data normalization removed a significant part of the subject-related component, but it did not cancel it completely. The remnant component can easily be observed in Figure 3, in the form of small clusters of samples that belong to the same individual.

### 5.2. Scalability

**Figure 5:**
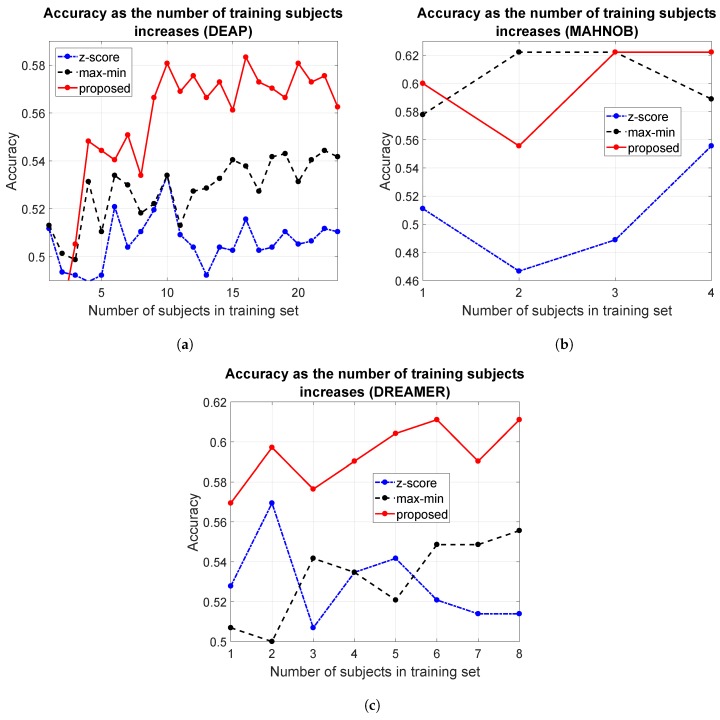
Classification accuracy as the number of training users is increased: (**a**) DEAP; (**b**) MAHNOB-HCI; and (**c**) DREAMER.

To further test the scalability and generalization capacity of the proposed method, we designed a second experiment that aimed to test how predictions improve as more subjects are incorporated into the training. As an example, Figure 5 shows the results obtained in the three repositories for different approaches, when using a Naive Bayes classifier to predict valence. The methods compared were the *z*-score normalization, the proposed data transformation and the subject-based scaling proposed in [33,65], in which features were scaled to the range [0,1]. The latter method is labeled as max-min in the figure.

In this plot, the classification accuracy reported for a number of training subjects *p* is the average of as many trials as subjects there are in the dataset. In each trial, a different subject was considered, and all his/her samples were included in the test set. The training set was composed of all samples from *p* subjects other than the test subject, chosen at random but maintained across the different algorithms to allow for a fair comparison.

Both the proposed normalization and the subject-based max-min scaling used in [33,65] showed better results when more subjects were used for learning. On the contrary, the *z*-score normalization did not seem to benefit from learning when the number of training subjects increased. The higher performance of the proposed data transformation can easily be observed in all databases. This shows up as a positive trend that implies a reliability increase as more users are incorporated into the model, and further supports the validity of inter-subject models when they are combined with a suitable transformation function that takes individual traits into consideration.

## 6. Conclusions

Subject-independent models fail to consider that the appraisal of one’s emotional state is strongly related to personal factors, such as one’s circumstances [39]. Subject-dependent models aim to tackle this weakness, but they do so at the cost of significantly increasing data collection needs [40,41]. This implies that they have to be individually trained for each user and hence cannot be used with previously unseen subjects.

In this paper, a mixed framework to support automatic emotion recognition is proposed. Unlike most typical subject-dependent modeling approaches, the method uses data from all users to build the model, and can be used to make predictions for previously unseen users in an adaptive way, increasing performance as more training data become available. We first show that the existence of an inherent subject-related component in the EEG signals is a major obstacle when attempting to build a user independent model that is simultaneously valid for all subjects. Then, we propose a subject-based normalization procedure that is able to reduce the magnitude of this component when using PSD features. This straightforward normalization procedure is not intended to be a solution to remove this component, but rather a demonstration of the potential benefits of reducing its magnitude. The removal of the subject-dependent component in the signal is indeed feature and problem dependent, and an optimum approach cannot be generalized at this stage. This implies that there is still room for improvement by designing other normalization methods that are more efficient at this task.

The impact of the proposed method goes beyond the construction of inter-subject models for emotion detection from EEG signals. First, the same principles can be exported to other sources of information other than EEG, e.g., physiological, audio, and video. Second, these principles are not limited to the particular problem of emotion recognition. On the contrary, the subject-related component is intrinsic to the signal, and it is present regardless of the problem context.

## Figures and Tables

**Table 1:** Summary of characteristics for the databases in the study.

Database	Subjects	Videos	Stimuli	Duration	Device	Channels	Sampling Frequency	Features
DEAP	32	40	Music	60 s	Biosemi	32	512 Hz *	230
			videos		Active II			
MAHNOB	27	20	Excerpts	34.9–117 s	Biosemi	32	512 Hz *	230
			from movies	(M = 81 s)	Active II			
DREAMER	23	18	Music	65–393 s	Emotive	14	128 Hz	105
			videos	(M = 199 s)	EPOC			

* downsampled to 256 Hz.

**Table 2:** Number of subjects and samples per subject in each dataset, after pre-processing.

	Valence		Arousal	
Database	Number of	Samples per	Number of	Samples
	Subjects	Subject	Subjects	per Subject
DEAP	24	32	16	32
MAHNOB	5	18	10	18
DREAMER	9	16	7	14

**Table 3:** Results obtained with a typical *z*-score normalization and with the proposed data transformation.

		Valence			Arousal		
		SVM	SVM	Naive	SVM	SVM	Naive
		Cubic	Radial	Bayes	Cubic	Radial	Bayes
DEAP	*z*-score	0.51	0.50	0.51	0.52	0.50	0.50
	proposed	0.54	0.58	0.57	0.54	0.56	0.55
MAHNOB	*z*-score	0.50	0.56	0.56	0.55	0.52	0.57
	proposed	0.51	0.65	0.65	0.59	0.61	0.62
DREAMER	*z*-score	0.50	0.52	0.51	0.55	0.53	0.50
	proposed	0.54	0.59	0.59	0.58	0.57	0.57

**Table 4:** Results of Friedman test on data reported in Table 3.

		Valence			Arousal		
		SVM	SVM	Naive	SVM	SVM	Naive
		Cubic	Radial	Bayes	Cubic	Radial	Bayes
DEAP	pairwise comparisons	480	480	480	320	320	320
	average rank *z*-score	1.65	1.73	1.71	1.57	1.78	1.78
	average rank proposed	1.35	1.27	1.29	1.43	1.22	1.22
	*p*-value	<10${}^{-10}$	<10${}^{-22}$	<10${}^{-18}$	0.01	<10${}^{-23}$	<10${}^{-23}$
MAHNOB	pairwise comparisons	100	100	100	200	200	200
	average rank *z*-score	1.61	1.82	1.84	1.58	1.84	1.70
	average rank proposed	1.39	1.18	1.16	1.42	1.16	1.30
	*p*-value	0.02	<10${}^{-10}$	<10${}^{-11}$	0.02	<10${}^{-21}$	<10${}^{-8}$
DREAMER	pairwise comparisons	180	180	180	140	140	140
	average rank *z*-score	1.66	1.81	1.82	1.54	1.66	1.73
	average rank proposed	1.34	1.19	1.18	1.46	1.34	1.27
	*p*-value	<10${}^{-4}$	<10${}^{-15}$	<10${}^{-17}$	0.31	<10${}^{-3}$	<10${}^{-7}$

**Table 5:** Results when using an intra-subject model, in the three databases.

	Valence			Arousal		
	SVM	SVM	Naive	SVM	SVM	Naive
	Cubic	Radial	Bayes	Cubic	Radial	Bayes
DEAP	0.62	0.64	0.61	0.55	0.54	0.59
MAHNOB	0.59	0.59	0.58	0.56	0.66	0.62
DREAMER	0.50	0.52	0.46	0.49	0.51	0.51

## Abbreviations

The following abbreviations are used in this manuscript:

DEAP	Database for Emotion Analysis using Physiological Signals
EEG	Electroencephalography
fMRI	Functional Magnetic Resonance Imaging
LDA	Linear Discriminant Analysis
PSD	Power Spectral Density
RBF	Radial Basis Function
SVM	Support Vector Machine
t-SNE	t-Distributed Stochastic Neighbor Embedding
